# Deep Learning-Driven Pathological Prediction of Lymph Node Metastasis in Patients with Head and Neck Squamous Cell Carcinoma Using Primary Whole Slide Images

**DOI:** 10.3390/cancers18060933

**Published:** 2026-03-13

**Authors:** Zaizai Cao, Zhe Chen, Jiangtao Zhong, Hengchao Chen, Ziming Fu, Zuning Shi, Jingyao Chen, Yajun Yu, Shuihong Zhou

**Affiliations:** 1 Department of Otolaryngology, The First Affiliated Hospital, College of Medicine, Zhejiang University, Hangzhou 310058, China; 1323006@zju.edu.cn (Z.C.); 1513022@zju.edu.cn (Z.C.); chenhengchao@126.com (H.C.); fzming16@zju.edu.cn (Z.F.); 3200105544@zju.edu.cn (Z.S.); 2 Facility for Histomorphology, Core Facilities, Zhejiang University School of Medicine, Hangzhou 310052, China; 0914255@zju.edu.cn (J.C.); 0922002@zju.edu.cn (Y.Y.)

**Keywords:** head and neck squamous cell carcinoma, lymph node metastasis, whole-slide image, deep learning, multiple instance learning, computational pathology

## Abstract

Background/Objectives: Accurate preoperative prediction of cervical lymph node metastasis (LNM) in head and neck squamous cell carcinoma (HNSCC) remains a major clinical challenge. This study aimed to develop a deep learning-based whole-slide image (WSI) model and an integrated nomogram to improve individualized LNM risk stratification. Methods: A total of 355 formalin-fixed paraffin-embedded (FFPE) WSIs and 282 frozen WSIs from the TCGA-HNSC cohort, along with 329 FFPE WSIs from an external institutional cohort, were retrospectively analyzed. Tumor regions were annotated and tiled into standardized patches. A dual-stage multiple instance learning framework was applied to generate WSI-level predictions. A pathological risk score (path-score) was derived and combined with clinical variables to construct a predictive nomogram. Results: The WSI-level model outperformed patch-level classifiers, with the logistic regression-based model achieving area under the curve (AUC) values of 0.821 in the internal validation cohort and 0.730 in the external cohort. The path-score was independently associated with LNM. The integrated nomogram further improved discrimination, yielding AUCs of 0.865 and 0.786 in the internal and external cohorts, respectively. Calibration and decision curve analyses demonstrated good agreement and meaningful clinical benefit. Conclusions: This deep learning-driven pathology nomogram provides a robust and clinically applicable tool for preoperative prediction of cervical lymph node metastasis in HNSCC.

## Simple Summary

Lymph node metastasis is one of the most important factors affecting treatment decisions and survival in patients with head and neck squamous cell carcinoma. However, accurately identifying patients at high risk before surgery remains challenging. In this study, we used digital pathology images of primary tumors and artificial intelligence to predict whether cancer had spread to cervical lymph nodes. By analyzing whole-slide images with a deep learning model and combining the results with basic clinical information, we developed a prediction tool that provides individualized risk estimates. Our model showed reliable performance in both internal and external patient cohorts and demonstrated potential clinical value for guiding neck management. This approach may help reduce unnecessary surgical procedures while ensuring timely treatment for patients at high risk of lymph node metastasis.

## 1. Introduction

Head and neck squamous cell carcinoma (HNSCC) is one of the most common malignancies worldwide, ranking as the seventh most prevalent cancer and accounting for significant cancer-related mortality. HNSCC represented approximately 4.5% of all newly diagnosed cancer cases globally [1]. HNSCC arises from the mucosal epithelium of the oral cavity, oropharynx, larynx, and hypopharynx, and comprises several pathological subtypes depending on the anatomic origin. Among these, squamous cell carcinoma of the oral cavity and larynx are the most frequent types, together constituting the majority of HNSCC cases. For patients with localized HNSCC, treatment strategies have evolved beyond traditional surgical resection and radiotherapy to incorporate a broader range of multimodal therapeutic options. Depending on tumor site, stage, and functional considerations, management may include surgery, radiotherapy, concurrent chemoradiotherapy, induction chemotherapy, and, more recently, immunotherapy with immune checkpoint inhibitors [2]. Early-stage HNSCC generally achieves favorable outcomes with single-modality treatment such as transoral surgery or definitive radiotherapy. However, patients with locoregionally advanced disease frequently require combined-modality therapy, and despite these intensified regimens, long-term survival remains suboptimal [3]. Recurrent or metastatic HNSCC continues to pose substantial therapeutic challenges, with 5-year overall survival largely limited [2,3].

Lymph node metastasis (LNM) is one of the most critical prognostic factors in HNSCC and represents a key step in tumor progression toward distant dissemination [4]. The presence of nodal involvement significantly reduces survival rates and increases recurrence risk [5], underscoring the need for accurate preoperative prediction and assessment of nodal status. Nevertheless, the clinical benefit and extent of elective neck dissection remain controversial, particularly in patients with clinically node-negative (cN0) disease [6,7]. Furthermore, micro-metastases may be missed during routine pathological examination, which would require serial sectioning and more exhaustive histopathological review [8].

Recently, with the rapid advancement of artificial intelligence (AI) in medical imaging, deep learning-based models have been applied to predict lymph node metastasis using histopathological features in various cancers, such as gastric [9], colorectal [10], prostate [11] and renal cell [12] cancers. Inspired by these developments, in this study, we developed a deep learning-based framework to predict lymph node involvement directly from whole slide images (WSIs) of primary HNSCC tumors.

To date, most preoperative LNM prediction models in HNSCC rely on radiological radiomics or clinical parameters [13,14], while WSI-based deep learning approaches remain limited and are mainly focused on survival outcomes. Our study represents one of the first large-scale investigations leveraging primary tumor WSIs with a two-stage MIL framework to directly predict cervical LNM and integrating pathological risk scores into a clinically interpretable nomogram.

## 2. Materials and Methods

### 2.1. Study Design and Ethical Approval

This retrospective study utilized publicly available datasets and institutional patient cohorts. WSIs and clinical information from the TCGA-HNSC cohort were retrieved through the Genomic Data Commons, which requires no additional ethical approval. The external validation cohort (China-HNSCC) was obtained from the First Affiliated Hospital of Zhejiang University, where ethical clearance was granted by the institutional review board. All WSIs were fully anonymized before analysis to ensure patient confidentiality, and no informed consent was required due to the retrospective design and use of de-identified data.

Generative artificial intelligence tools were used only for language polishing and did not affect the scientific content.

### 2.2. Patient Cohorts and Dataset Partition

In this study, WSIs of two large cohorts were collected, and an LNM label was assigned to each WSI based on the patient’s pathology report. The first cohort (TCGA HNSC), retrieved from The Cancer Genome Atlas, comprised 541 frozen tissue slides and 450 formalin-fixed, paraffin-embedded (FFPE) sections diagnosed as HNSCC at stages I to IV. Slides were excluded based on: 1. Insufficient tumor content (<50% tumor tissue). 2. Severe staining artifacts (e.g., overstaining, ink marks, tissue folds). 3. Frozen section artifacts affecting tissue integrity. 4. Missing clinical or LNM data. To avoid repeated sampling bias, only one representative whole-slide image per patient was included for model development and evaluation. Finally, 355 FFPE WSIs and 282 frozen WSIs fulfilled inclusion criteria. FFPE WSIs were partitioned at the patient level into the following categories: Training set, with 284 slides (80%), and Internal validation set, with 71 slides (20%). Frozen WSIs were used only for external robustness assessment. The external validation cohort was collected from the first affiliated hospital of Zhejiang University (China-HNSCC cohort) and consisted of 329 FFPE sections diagnosed with HNSCC of all stages. All WSIs used in this study were obtained from surgically resected primary tumor specimens. FFPE slides were prepared following routine postoperative pathological processing, while frozen-section WSIs were derived from intraoperative frozen specimens in the TCGA-HNSC cohort. Although pathological images were generated after tumor resection, the histopathological characteristics captured by WSIs reflect intrinsic tumor biological properties that exist prior to surgery.

Within the training cohort, a five-fold cross-validation strategy was applied for model optimization and hyperparameter tuning. The internal validation cohort was used for independent performance assessment, and the independent China-HNSCC cohort and frozen tissue slides were used for external validation to evaluate model generalizability. Importantly, cross-validation was strictly performed within the training cohort only, without any overlap of patients across different subsets.

### 2.3. ROI Delineation, Tiling, and Data Preprocessing

All WSIs were digitalized with a 20× objective lens with a predefined pixel resolution (~0.5 μm/pixel). In order to reduce the influence of unrelated areas and alleviate the workload of the classification method, regions of carcinoma (ROIs) on WSIs were manually annotated by expert pathologists, according to the following rules: (1) the tumor cells should occupy more than 80% of a ROI, i.e., the interstitial component is less than 20%, and (2) obvious interfering factors, including creases, bleeding, necrosis and blurred areas, should be excluded. The annotation was performed using QuPath-0.3.2.

Given the extremely large image size (typically 100,000 × 50,000 pixels) of a WSI, the WSIs were subsequently tiled into 512 × 512 patches. Only patches with a greater than 80% overlap with the carcinoma ROI were used for the following analysis. To minimize inter-slide staining variability, the following preprocessing steps were applied: 1. Macenko color normalization; 2. Z-score pixel standardization.

### 2.4. Multiple Instance Learning (MIL)-Based Deep Learning Pipeline

**Figure 1:**
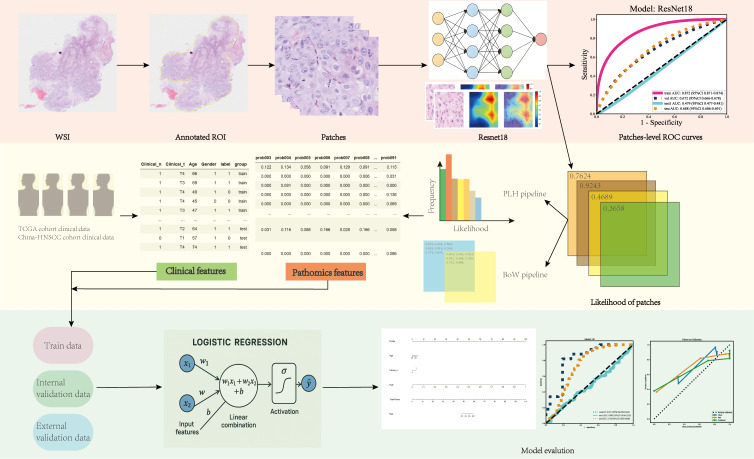
The workflow of artificial intelligence model development. Hematoxylin and eosin-stained slices of head and neck cancer were collected for digital whole slide scanning. Next, ResNet-18 was employed to develop a patch-level artificial intelligence model. Two independent multiple instance learning (MIL) pipelines, namely the Patch Likelihood Histogram (PALHI) pipeline and the Bag of Words (BoW) pipeline, were employed to extract whole slide image (WSI)-level features. The derived WSI features were integrated with clinical variables to construct a nomogram model. Model performance was evaluated in the internal and external validation cohorts, with additional assessment conducted on a frozen-slide cohort to examine model generalizability and potential domain shift across different tissue processing modalities.

We employed the previously reported Ensembled Patch Likelihood Aggregation (EPLA) model [15] architecture to train the model in the TCGA-HNSC cohort training set (split in an 8:2 ratio for training and testing). The model consists of two consecutive stages: patch-level prediction and whole-slide image-level prediction. The workflow of the AI model construction is shown in Figure 1.

During the patch-level prediction, a residual convolutional neural network (ResNet-18) was trained to compute the patch likelihood in a MIL paradigm where the patches were assigned with the WSI’s label. Binary cross-entropy (BCE) loss was utilized to optimize the network using a mini-batch gradient descent method. Model training was performed using SGD (initial learning rate = 0.01, batch size = 64, epochs = 50). Input tiles were normalized with ImageNet mean and standard deviation, and weights were initialized from ImageNet-pretrained checkpoints. No class-balanced sampling was applied (batch_balance = False). Training was conducted on one GPU with 16 data-loader workers. Hyperparameters, including learning rate, batch size, number of training epochs, and optimizer parameters, were tuned based on validation performance, with AUC used as the primary selection criterion.

Two independent MIL methods were developed to aggregate the patch likelihoods: the Patch Likelihood Histogram (PALHI) pipeline and the Bag of Words (BoW) pipeline, which were inspired by the histogram-based method and the vocabulary-based method, respectively. In PALHI, a histogram of the occurrence of the patch likelihood was applied to represent the WSI, whereas in BoW, each patch was mapped to a TF-IDF floating-point variable, and a TF-IDF feature vector was computed to represent the WSI. Traditional machine learning classifiers were then further trained using these feature vectors to predict the MS status for each WSI. Here, Extreme Gradient Boosting (XGboost), a kind of gradient boosted decision tree, was employed in the PALHI pipeline. Naïve Bayes (NB) was used in the BoW pipeline. During the training of the WSI-level classifier, the hyperparameters were determined based on cross-validation on the training set, using the whole slide image-level receiver operating characteristic (ROC) area under the curve (AUC) as the performance metric. During WSI-level prediction, the outputs of the PALHI and BoW classifiers were then ensembled to obtain the final prediction.

### 2.5. Development of the Path-Score and Multimodal Nomogram

To enhance interpretability, we extracted WSI-level features from the MIL ensemble and used LASSO regression to derive a path-score, representing the quantitative pathological risk of LNM. Next, three independent predictors were identified via multivariate logistic regression: clinical N stage, age and path-score. Based on the regression coefficients of these variables, we constructed a combined clinical–pathomics nomogram for individualized prediction of LNM probability.

### 2.6. Model Evaluation and Statistical Analysis

Model discrimination was assessed using AUC, sensitivity, specificity, accuracy, and 95% confidence intervals (CIs). Evaluations were performed on the Internal validation cohort (TCGA-FFPE); External validation cohort (China-HNSCC), and frozen-section cohort (TCGA-frozen) for generalizability. Patch-level and WSI-level performances were compared to confirm the benefit of MIL aggregation. Calibration performance was evaluated using calibration curves and Hosmer–Lemeshow goodness-of-fit tests. Across clinically relevant threshold probability ranges, we evaluated the net benefit to quantify the practical value of the clinical model, the path-score model, and the integrated nomogram, and visualized these results using decision curve analysis (DCA) curves. Univariate and multivariate logistic regression analyses were conducted to identify predictors of LNM. Variables with *p* < 0.05 in univariate analysis were included in multivariate models. All analyses were performed using Python (version 3.9) and the Onekey platform (version 4.10.27).

## 3. Results

### 3.1. Performance of Patch-Level Models

**Figure 2:**
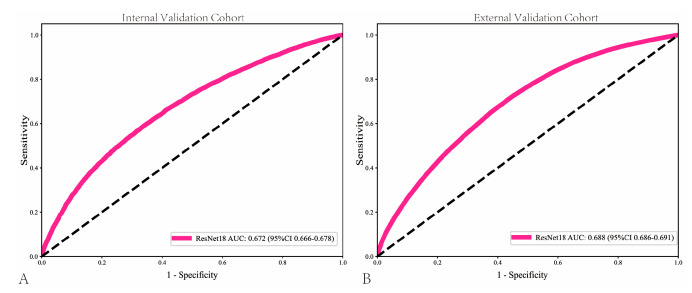
Patch-level receiver operating characteristic (ROC) curves in the internal validation (**A**) and external validation (**B**) cohorts.

The pathomics-based model named EPLA was developed in the training set of the TCGA-HNSC cohort (8:2 for training and test), which consisted of two consecutive stages: patch-level prediction and WSI-level prediction. Briefly, a WSI was annotated to delineate the region of ROI. The ROI was tiled into patches, which were subsequently fed to ResNet-18 to obtain the patch-level LNM prediction. Figure 2 presents the AUC for the model. The patch-level AUC for predicting LNM in the internal validation cohort and testing cohort was 0.672 (95% CI: 0.666–0.677) and 0.688 (95%CI: 0.686–0.691), respectively. The Sensitivity in the internal validation cohort and testing cohort was 0.565 and 0.672, respectively, while the Specificity was 0.686 and 0.601, respectively.

### 3.2. Performance of WSI-Level MIL Models

**Figure 3:**
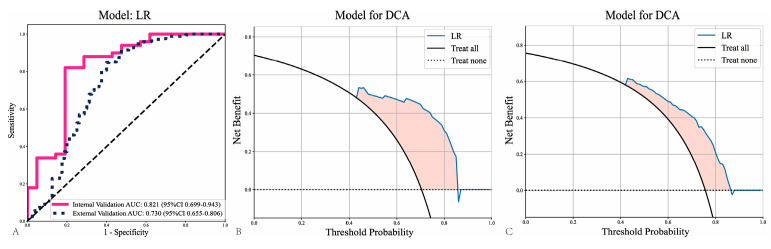
Receiver operating characteristic (ROC) curves of the whole slide image (WSI)-level logistic regression (LR) model in the internal and external validation cohorts (**A**). Decision curve analysis (DCA) illustrating the net benefit of the LR model across a range of threshold probabilities in the internal validation cohort (**B**) and the external validation cohort (**C**).

To further assess the model’s performance, two independent MIL pipelines (PALHI and BoW) were trained to integrate multiple patch-level predictions. Patches were aggregated into WSI levels to assess the performance of the models. Compared with patch-level predictions, there were better performances from all WSI-level machine learning models on the internal validation cohort and external validation cohort (Table 1). Among all models (Logistic Regression, LR; Support Vector Machine, SVM; RF, Random Forest), the LR model showed the best efficiency in the internal validation cohort (AUC = 0.821; 95%CI: 0.699–0.943) and the China-HNSCC cohort (AUC = 0.730; 95%CI: 0.655–0.806) (Figure 3A). This demonstrates that the WSI-level approach leads to better prediction performance compared to patch-level predictions. As shown in Figure 3B,C, decision curve analyses showed good clinical benefit. Our study calculated the path-score as a linear combination of the nonzero coefficient features identified through the LASSO model.

### 3.3. Evaluation of Model Generalizability in Frozen Sections

To further assess model generalizability across different tissue processing modalities, the WSI-level analytical framework trained on FFPE slides was applied to frozen-section WSIs from the TCGA-HNSC cohort. In this frozen cohort, the WSI-level model achieved an AUC of 0.485, indicating a marked decline in predictive performance compared with FFPE-based cohorts (Supplementary Figure S1). This result suggests limited transferability of FFPE-trained models to frozen tissue slides.

The reduced performance observed in frozen-section WSIs is likely attributable to the domain shift introduced by different tissue processing protocols. Frozen sections are more susceptible to preparation-related artifacts, including ice crystal formation, tissue deformation, and staining inconsistency, which may substantially alter image texture and color distribution compared with FFPE slides. As the model was primarily trained on FFPE WSIs, this discrepancy inevitably affected cross-domain generalization.

### 3.4. Univariate and Multivariate Analyses of Clinical Variables

Univariate logistic regression analysis was conducted to evaluate the association between clinical variables and LNM risk (Table 2). In the univariate analysis, clinical N stage showed the strongest association with LNM (OR = 5.591, 95% CI: 3.819–8.183, *p* < 0.01), followed by clinical T stage (OR = 1.194, 95% CI: 1.091–1.306, *p* < 0.01), age (OR = 1.005, 95% CI: 1.001–1.008, *p* < 0.05), and gender (OR = 1.405, 95% CI: 1.111–1.777, *p* < 0.05).

After adjustment for covariates in the multivariate logistic regression model, only clinical N stage (OR = 12.112, 95% CI: 7.382–19.866, *p* < 0.01) and age (OR = 0.987, 95% CI: 0.978–0.997, *p* < 0.05) remained independently associated with LNM risk. Clinical N stage remained the strongest independent predictor of lymph node metastasis, indicating that patients with radiologically positive lymph nodes had more than a ten-fold higher risk of pathological metastasis compared with clinically node-negative patients. This finding highlights the dominant role of nodal imaging assessment in preoperative risk stratification. Age showed a modest but statistically significant association with lymph node metastasis, suggesting a small inverse relationship between age and metastatic risk.

### 3.5. Development and Validation of the Integrated Nomogram

A combined nomogram incorporating three independent predictors was developed: clinical N stage, age, and path-score (Supplementary Figure S2). The nomogram utilizes the regression coefficients of these variables to calculate a total score, with individual points assigned to each variable on the basis of its contribution. The nomogram assigns points to each factor using a point scale. These points are then combined to predict LNM probability. The nomogram model performance was compared to the path-score model and a model based solely on clinical features.

**Figure 4:**
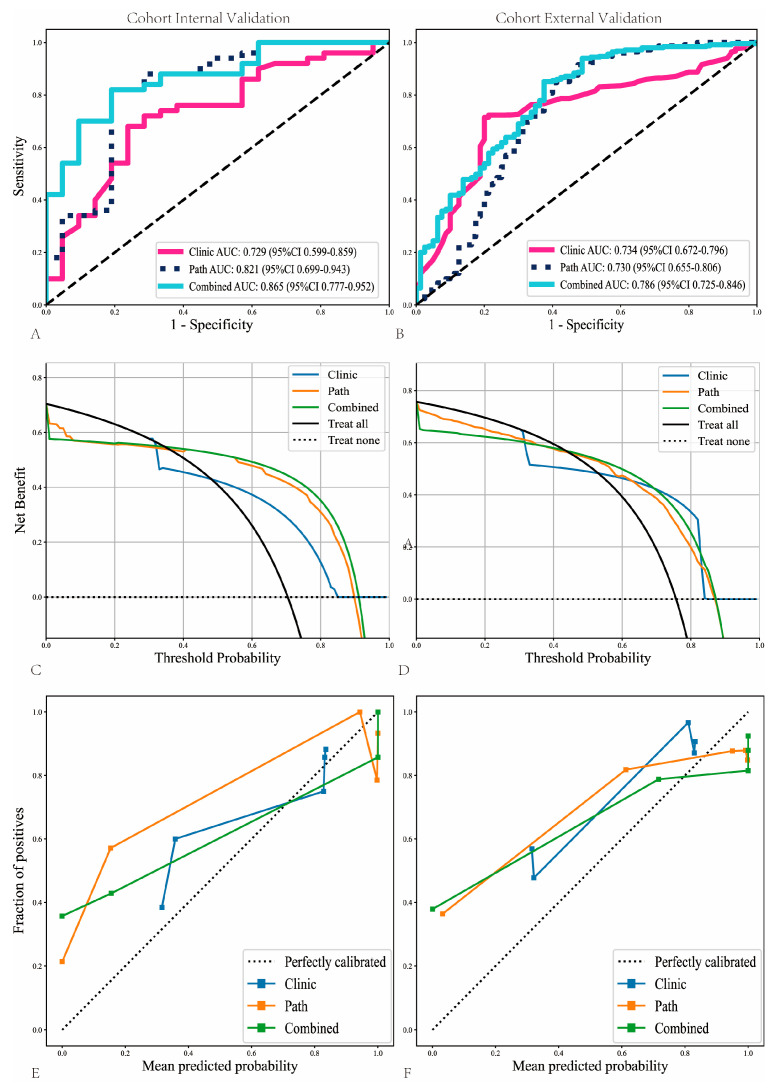
Performance comparison of the integrated nomogram, path-score model, and clinical model in the internal and external validation cohorts. Receiver operating characteristic (ROC) curves demonstrating the discriminative performance of the three models in the internal validation cohort (**A**) and the external validation cohort (**B**). Decision curve analysis (DCA) illustrating the net benefit across a range of threshold probabilities in the internal (**C**) and external (**D**) validation cohorts. Calibration curves showing the agreement between predicted and observed probabilities for the three models in the internal (**E**) and external (**F**) validation cohorts.

Within the internal validation cohort, the nomogram achieved the highest AUC of 0.865 (95% CI: 0.777–0.952) outperforming the path-score model (AUC = 0.821, 95% CI: 0.699–0.943) and significantly surpassing the clinical model (AUC = 0.729, 95% CI: 0.599–0.859) (Figure 4A). This superior performance was replicated in the external validation cohort, where the nomogram elucidated an AUC of 0.786 (95% CI: 0.725–0.846) in comparison to 0.730 (95% CI: 0.655–0.806) for the path-score model and 0.734 (95% CI: 0.672–0.796) for the clinical model (Figure 4B).

Beyond discrimination performance, decision curve analysis (DCA) further demonstrated the clinical utility of the nomogram. Within both the internal and external validation cohorts, the nomogram consistently yielded the highest net benefit across a wide range of threshold probabilities compared with the clinical-only and path-score models (Figure 4C,D), indicating superior decision-making value in predicting lymph node metastasis.

Model calibration also showed favorable agreement between predicted and observed probabilities. In the internal cohort, the nomogram exhibited the closest alignment to the ideal calibration line, whereas the clinical and path-score models showed noticeable deviations at higher predicted probabilities (Figure 4E). Similar trends were observed in the external cohort, where the nomogram maintained stable calibration performance with reduced prediction bias (Figure 4F).

Collectively, these results indicate that the integrated nomogram not only enhances discriminatory accuracy but also provides better clinical applicability and more reliable risk estimation compared with single-modality models.

## 4. Discussion

In this multicenter retrospective study, we developed a deep learning-driven computational pathology framework to predict cervical lymph node metastasis (LNM) directly from primary whole slide images (WSIs) in head and neck squamous cell carcinoma (HNSCC). By leveraging a dual-stage multiple instance learning (MIL) architecture and deriving a WSI-based path-score that was subsequently integrated with clinical variables into a nomogram, we achieved robust discrimination, good calibration, and meaningful net benefit across both internal and external cohorts. These findings support the concept that routine histomorphologic patterns of the primary tumor contain rich information about metastatic propensity that extends beyond conventional clinicopathologic assessment alone [9,11,12,16,17,18].

Cervical LNM remains one of the most important adverse prognostic factors in HNSCC, being associated with higher locoregional failure and reduced survival [1,4,7]. Occult nodal disease is not uncommon in clinically node-negative (cN0) patients, especially in supraglottic and other high-risk subsites, and several series have highlighted its negative impact on outcomes [7,16,17,19]. Consequently, there is ongoing controversy around the management of the cN0 neck. Randomized and observational data in oral cavity and laryngeal cancer indicate that elective neck dissection (END) can improve disease control and survival but inevitably subjects a substantial proportion of truly node-negative patients to unnecessary morbidity [6]. In this context, a non-invasive and accurate tool for individualized preoperative LNM risk stratification could refine indications for END, sentinel node biopsy, or intensified surveillance.

Previous prediction models for LNM in HNSCC have largely relied on clinical and conventional histopathologic variables. T category, supraglottic involvement, tumor budding, lymph vascular invasion, and other factors have shown good discriminatory performance and calibration for estimating cervical LNM or occult nodal disease [20,21]. More recently, radiomics and deep learning models based on CT or dual-energy CT (DECT) have demonstrated additional value. Zhao et al. showed that CT-based radiomics significantly improved preoperative prediction of cervical LNM in LSCC compared with size-based criteria alone [13]. Zhang et al. further developed a DECT iodine-map radiomics nomogram for HNSCC that provided accurate and clinically useful LNM prediction across centers [14]. DECT- or spectral CT-based models specifically tailored to LSCC have also been reported, underscoring the promise of advanced imaging biomarkers to complement routine evaluation [22].

In contrast, our study exploits only routine hematoxylin–eosin WSIs of the primary tumor, which are generated for virtually all surgically treated patients and are increasingly digitized. Our MIL-based framework is conceptually aligned with prior computational pathology work in gastric, colorectal, prostate, and renal cancers, where deep learning applied to primary tumor or lymph node WSIs successfully captured metastatic behavior and prognostic information that can be difficult for human observers to quantify consistently [9,10,11,12,16]. Importantly, we demonstrate that WSI-level MIL models substantially outperform patch-level classifiers, emphasizing the importance of global tumor context and heterogeneity rather than isolated tiles.

Within HNSCC, most AI work related to nodal disease has focused either on cross-sectional imaging of lymph nodes or on analysis of the resected lymph nodes themselves. Tang et al. proposed a two-step deep learning system for HE-stained lymph node sections in HNSCC and reported high sensitivity for detecting metastases, suggesting that deep learning can augment routine pathology of nodal specimens [23]. In the imaging domain, multiple radiomics and deep learning studies have explored CT, DECT, MRI, and even ultrasound for predicting nodal status and extranodal extension in HNSCC, often achieving performance comparable to expert radiologists [13,22,24,25]. Ultrasound-based radiomics and neural network models for cervical lymph nodes are also emerging, potentially useful in the preoperative staging clinic [24]. By focusing instead on primary tumor WSIs, our framework addresses a complementary question: whether the primary tumor “phenotype” alone encodes sufficient information to infer nodal risk, independent of nodal imaging.

A key contribution of this work is the derivation of a path-score from LASSO-selected WSI-level features and its integration with clinical predictors in a combined nomogram. Our multivariable analysis confirmed that the path-score was an independent predictor of LNM alongside clinical N stage and age, while clinical T stage lost significance after adjustment. This suggests that deep learning-derived pathomic features can partially compensate for the limited discriminatory power of traditional anatomic staging, capturing aspects of tumor architecture, stromal response, and microenvironment that are not included in TNM but are biologically linked to metastatic spread [26]. The combined nomogram consistently outperformed both the clinical-only and path-score-only models in internal and external cohorts and yielded the highest net benefit across clinically relevant threshold probabilities on decision-curve analysis, supporting its potential utility for individualized neck management.

Another strength of our study is the external validation across distinct data sources, including an independent institutional FFPE cohort. Despite variability in staining protocols, scanners, and patient populations, the nomogram maintained good discrimination and calibration. These results are consistent with broader experience in deep-learning WSI analysis, where appropriate regularization and domain-robust training strategies can yield reasonably generalizable models [11,12,27]. Nevertheless, we observed a marked drop in performance when directly applying the FFPE-trained framework to frozen sections. Frozen tissue introduces a substantial domain shift due to different morphology and staining characteristics [28,29]. This finding underscores the importance of stain- and domain-invariant methods, or explicit domain adaptation, before deploying computational pathology models across slide types or institutions.

Interpretability and biological plausibility are critical for clinical translation. Although MIL provides an efficient weakly supervised approach, it does not explicitly label the morphological determinants of high path-score. Recent work in interpretable deep learning for WSI-based LNM prediction in gastric cancer, as well as review articles on explainable WSI models, has emphasized the value of attention maps and concept-based analyses to bridge the gap between AI predictions and human pathology reasoning [17]. In future work, correlating high-attention tiles in our model with specific features—such as tumor budding, lymphovascular invasion, perineural invasion, immune cell density, or stromal reaction—could both enhance trust and yield new insights into the metastatic biology of HNSCC. Linking WSI features with spatial transcriptomics or multiplex immunohistochemistry may further elucidate how morphological patterns relate to underlying gene expression and immune contexture [30].

This study has several limitations. First, it is retrospective and subject to selection bias, with heterogeneous treatment strategies and follow-up, particularly in the external cohort. Prospective validation in well-annotated, contemporary HNSCC cohorts is essential to confirm reproducibility and assess clinical impact. Second, important biological variables such as HPV status, detailed patterns of perineural invasion, and systemic therapy regimens were not uniformly available and therefore could not be incorporated into the nomogram. Multi-modal fusion approaches that combine WSIs with radiomics, genomics, and immunologic biomarkers may further enhance prognostic and predictive performance. Third, although our decision-curve analysis suggests potential benefit for decision-making, we did not simulate specific clinical pathways (e.g., thresholds for recommending END) or quantify cost–benefit trade-offs; such analyses, together with pragmatic clinical trials, will be necessary to understand real-world utility. Finally, in this study, ROIs were annotated to prioritize tumor-dominant regions in order to reduce background noise and improve feature learning stability, which is a common practice in MIL-based computational pathology. However, we acknowledge that tumor stroma and the tumor–stroma ratio are important prognostic factors in HNSCC, and our ROI strategy may limit direct modeling of stromal components and microenvironmental heterogeneity. Future work will incorporate multi-region sampling and tumor–stroma interface modeling to jointly capture tumor morphology and microenvironmental features, thereby enhancing predictive performance and biological interpretability.

From a clinical translation perspective, our findings indicate that the current model is most suitable for FFPE-based pathological scenarios and requires further optimization before direct deployment on frozen-section images. Importantly, although the WSIs analyzed in this study were obtained from postoperative specimens, the prediction target represents the inherent metastatic potential of the primary tumor, which is determined before surgery. Therefore, this framework does not contradict the concept of preoperative risk stratification. In future studies, the proposed pipeline can be extended to biopsy or endoscopic specimens to enable true preoperative individualized lymph node metastasis risk assessment.

## 5. Conclusions

In conclusion, our study demonstrates that deep learning-driven WSI analysis offers a powerful and scalable approach for predicting lymph node metastasis in HNSCC. The proposed multimodal nomogram integrates pathomics with key clinical factors, resulting in superior predictive accuracy, robust generalizability, and meaningful clinical utility. This work provides a foundation for future translational applications of computational pathology to precision oncology in head and neck cancers.

## Figures and Tables

**Table 1:** Performance comparison of the prediction models in the internal and external validation cohorts.

Model	Accuracy	AUC	95% CI	Sensitivity	Specificity	Cohort
LR	0.803	0.821	0.699–0.943	0.800	0.810	Internal validation cohort
LR	0.784	0.73	0.655–0.806	0.843	0.600	External validation cohort
SVM	0.789	0.779	0.641–0.917	0.780	0.810	Internal validation cohort
SVM	0.836	0.710	0.630–0.790	0.924	0.562	External validation cohort
RF	0.732	0.753	0.618–0.888	0.700	0.810	Internal validation cohort
RF	0.666	0.648	0.573–0.723	0.711	0.525	External validation cohort

LR: Logistic Regression; SVM: Support Vector Machine; RF: Random Forest.

**Table 2:** Univariate and multivariate logistic regression analyses of clinical variables associated with lymph node metastasis.

	Univariate Logistic Regression			Multivariate Logistic Regression		
Characteristics	OR	95% CI	*p*	OR	95% CI	*p*
Clinical N Stage	5.591	3.819–8.183	<0.01	12.112	7.382–19.866	<0.01
Clinical T Stage	1.194	1.091–1.306	<0.01	0.960	0.748–1.231	0.786
Age	1.005	1.001–1.008	<0.05	0.987	0.978–0.997	<0.05
Gender	1.405	1.111–1.777	<0.05	1.120	0.680–1.842	0.709

95% CI, 95% confidence interval; OR, odds ratio.

## Data Availability

The TCGA-HNSC datasets analyzed in this study are publicly available from The Cancer Genome Atlas (TCGA) via the Genomic Data Commons portal. The institutional pathological whole-slide images used in this study are not publicly available due to ethical and privacy restrictions, but are available from the corresponding author upon reasonable request and with appropriate ethical approval.

## Supplementary Materials

The following supporting information can be downloaded at: https://www.mdpi.com/article/10.3390/cancers18060933/s1. Supplementary Figure S1: Receiver operating characteristic (ROC) curves in the frozen cohort. Supplementary Figure S2: Nomogram for predicting lymph node metastasis in head and neck squamous cell carcinoma.

## Abbreviations

The following abbreviations are used in this manuscript:

AI	Artificial intelligence
AUC	Area under the receiver operating characteristic curve
BoW	Bag of words
CI	Confidence interval
DCA	Decision curve analysis
DL	Deep learning
FFPE	Formalin-fixed paraffin-embedded
GDC	Genomic Data Commons
HNSCC	Head and neck squamous cell carcinoma
IRB	Institutional Review Board
LASSO	Least absolute shrinkage and selection operator
LNM	Lymph node metastasis
LR	Logistic regression
MIL	Multiple instance learning
NB	Naïve Bayes
OR	Odds ratio
PALHI	Patch likelihood histogram
ROC	Receiver operating characteristic
ROI	Region of interest
RF	Random forest
SVM	Support vector machine
TCGA	The Cancer Genome Atlas
WSI	Whole-slide image
