# Supplementary figures and images for: Deep Learning-Driven Pathological Prediction of Lymph Node Metastasis in Patients with Head and Neck Squamous Cell Carcinoma Using Primary Whole Slide Images
Source: Cancers (Basel). 2026 Mar 13;18(6):933. doi: 10.3390/cancers18060933 (PMC13025184; doi:10.3390/cancers18060933)

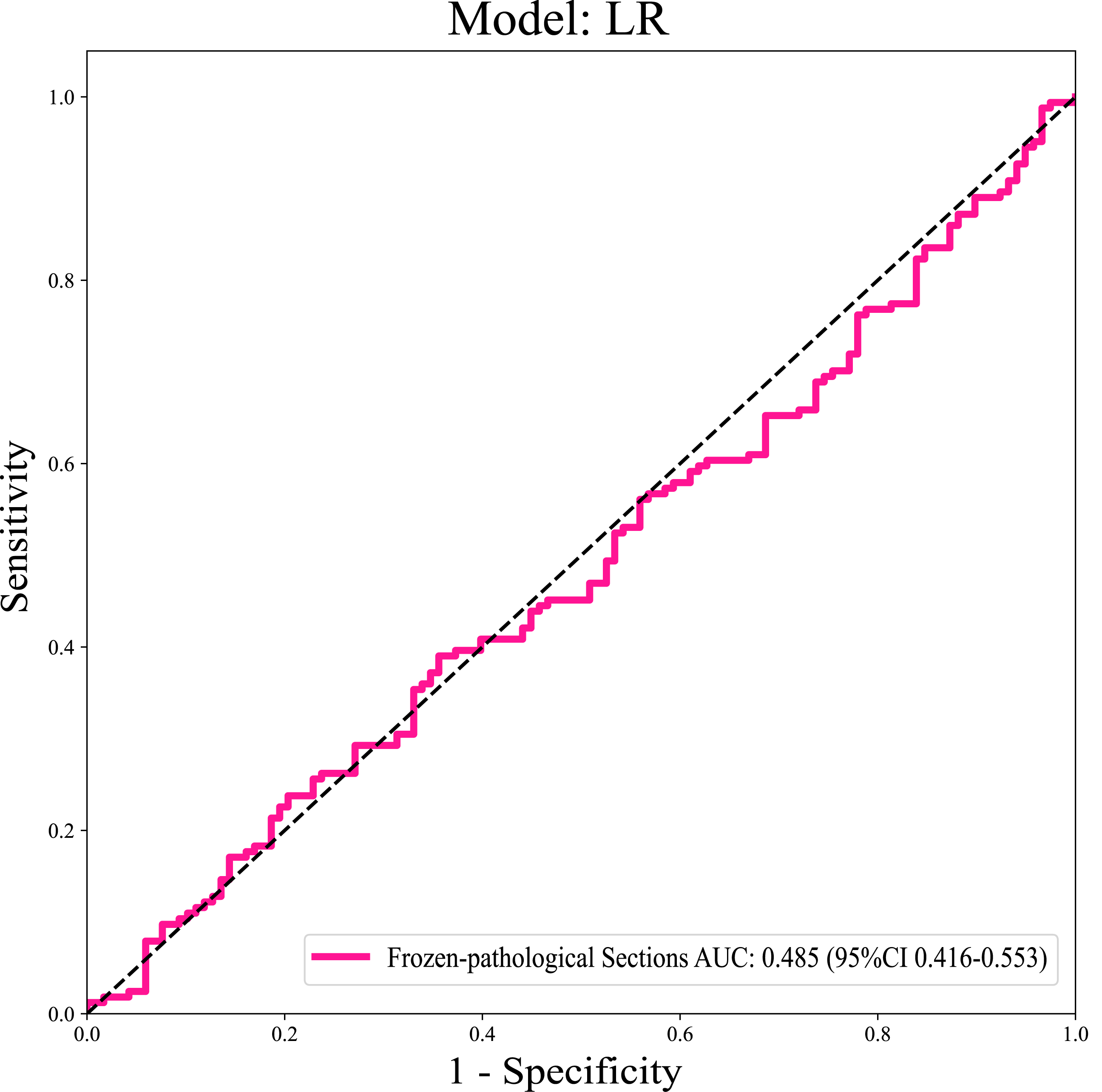

Supplement: Supplementary file 1 [file cancers-18-00933-s001.zip › Supplementary Figure S1.tif]
